# Potential applications and mechanisms of natural products in mucosal-related diseases

**DOI:** 10.3389/fimmu.2025.1594224

**Published:** 2025-04-30

**Authors:** Wang Lin, Xie Ruishi, Xu Caijiao, Luo Haoming, Hua Xuefeng, Yao Jiyou, Lu Minqiang, Zhou Shuo, Zhu Ming, Li Dongyang, Fang Xiaoxue

**Affiliations:** ^1^ Changchun University of Chinese Medicine, Changchun, China; ^2^ School of Pharmacy, Changchun University of Chinese Medicine, Changchun, China; ^3^ The First People’s Hospital of Guangzhou, Department of Hepatobiliary and Pancreatic Surgery, Guangzhou, China

**Keywords:** mucous barrier, natural products, immune regulation, inflammation inhibition, gut microbiota modulation

## Abstract

The mucosal barrier serves as a crucial defense against external pathogens and allergens, being widely distributed across the respiratory, gastrointestinal, urogenital tracts, and oral cavity. Its disruption can lead to various diseases, including inflammatory bowel disease, asthma, urinary tract infections, and oral inflammation. Current mainstream treatments for mucosa-associated diseases primarily involve glucocorticoids and immunosuppressants, but their long-term use may cause adverse effects. Therefore, the development of safer and more effective therapeutic strategies has become a focus of research. Natural products, with their multi-target and multi-system regulatory advantages, offer a promising avenue for the treatment of mucosal diseases. This review summarizes the potential applications of natural products in diseases of mucosal barrier dysfunction through mechanisms such as immune modulation, inflammation inhibition, tight junction protein restoration, and gut microbiota regulation, with the aim of providing insights for the exploration of novel therapeutic strategies.

## 1 Introduction

The mucosa serves as the body’s first line of defense against external pathogens and allergens and is widely distributed throughout the respiratory, gastrointestinal, genitourinary, and oral cavities (1). Far beyond a passive physical barrier, mucosa harbors a sophisticated immune network, including mucosa-associated lymphoid tissue (MALT) and diverse immune cells, which collectively regulate both innate and adaptive immune responses. Under homeostatic conditions, the structural integrity and immunological function of the mucosa are critical for maintaining host health and internal environmental stability (2).

However, various internal and external factors—such as infections, inflammatory responses, oxidative stress, medication use, and poor dietary habits—can impair mucosal barrier function, potentially leading to a range of chronic diseases. In the digestive system, intestinal mucosal damage can increase permeability, disrupt the microbiota, and trigger immune activation, all of which contribute to the pathogenesis of inflammatory bowel disease and food allergies. In the respiratory tract, mucosal damage increases the risk of asthma and chronic obstructive pulmonary disease and impairs respiratory function (3). In the urinary tract, mucosal damage predisposes individuals to urinary tract infections and impairs excretory function (4). Similarly, in the oral cavity, damage to the mucosal microenvironment is closely associated with conditions such as candidiasis and recurrent oral ulcers (5). Currently, commonly used clinical treatments—such as aminosalicylates, corticosteroids, and immunosuppressants—may alleviate symptoms in the short term, but long-term use often leads to drug resistance, hormone dependence, or systemic side effects, thereby limiting their therapeutic potential (6). Therefore, the development of novel, safe and effective therapeutics for mucosal diseases is urgently needed.

Natural products have unique advantages, including multi-system, multi-target, and multi-mechanism effects, providing new research directions for the treatment of mucosa-associated diseases (7). Numerous studies have demonstrated that natural products such as polysaccharides, alkaloids, and polyphenols can promote mucosal barrier repair through various mechanisms, including regulation of immune cell function, reduction of inflammatory cytokine levels, and enhancement of tight junction protein expression (8). In addition, there is increasing evidence of interactions between natural products and the host microbiota (9). Many natural products can modulate the composition of the gut microbiota, increase the abundance of short chain fatty acid (SCFA)-producing bacteria, and increase butyrate levels, thereby providing essential nutrients to intestinal epithelial cells and facilitating mucosal barrier repair. In addition, some metabolites of natural products exert protective effects on the mucosal barrier through mechanisms similar to those of their parent compounds.

These findings highlight the potential of natural products in the treatment of mucosal diseases. Although significant progress has been made in exploring the use of natural products for mucosa-related disease interventions, their mechanisms of action remain incompletely understood. Target specificity in different disease models, structure-function relationships, and interactions with host microecology require further investigation. This review aims to systematically summarize the latest advances in the application of natural products to various mucosa-associated diseases, with a focus on their core mechanisms in modulating mucosal immunity, repairing mucosal structures, and maintaining microecological balance, and discusses their clinical significance.

## 2 Immune system and mucosal barriers

### 2.1 Intestinal mucosal barrier

#### 2.1.1 Composition and barrier function of intestinal mucosa

**Figure 1:**
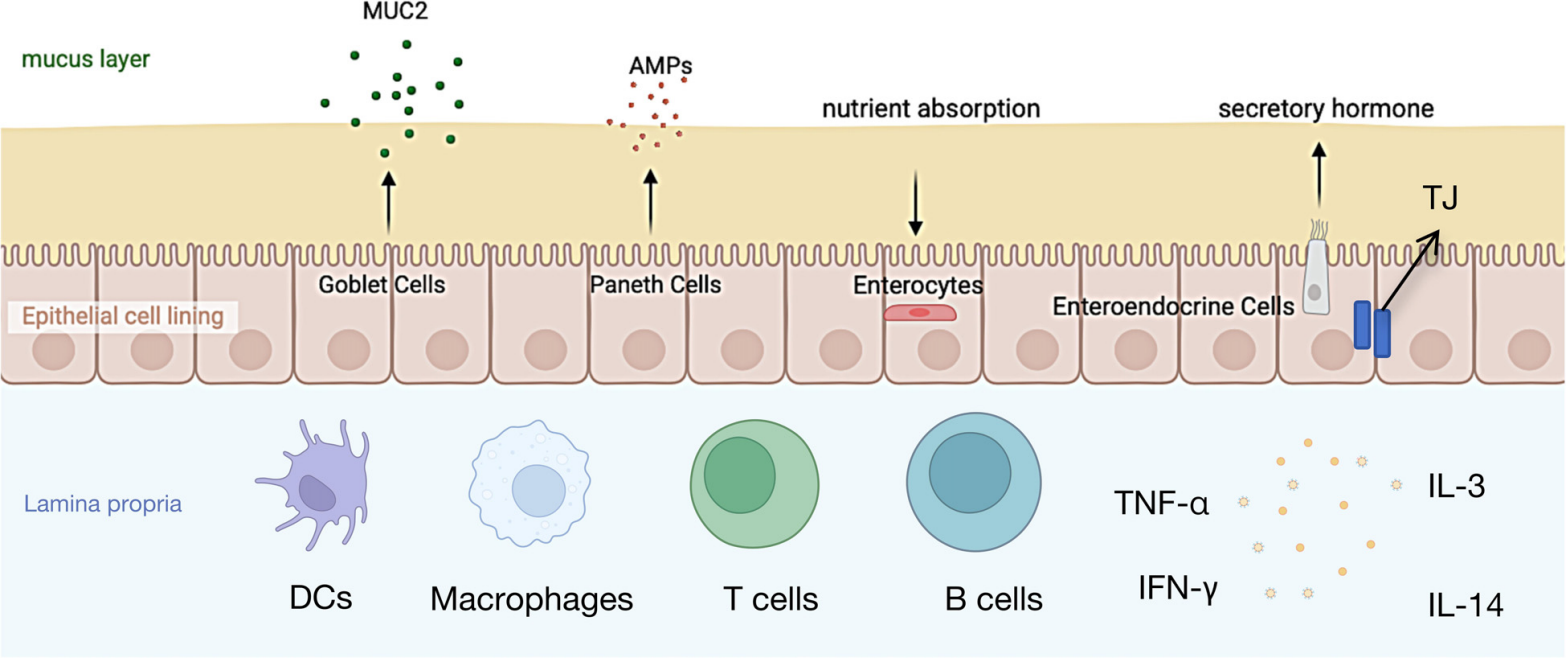
The structure of the intestinal mucosal barrier.

The intestinal mucosa serves as a critical immune barrier, not only facilitating nutrient absorption and providing an interactive surface for commensal microbes, but also preventing the invasion of harmful substances (10). The intestinal microbiota is comprised of several components, including an outer mucus layer containing commensal microbes, antimicrobial proteins (AMPs), and secretory immunoglobulin A (sIgA); a central monolayer of specialized epithelial cells; and an inner lamina propria housing innate and adaptive immune cells such as T cells, B cells, macrophages, and dendritic cells (11, 12) (**Figure 1**).

The mucus layer serves as the first physical barrier against external molecules entering the intestinal lumen (11), Its primary components are highly glycosylated mucins (13), which play a crucial role in protecting the immune system and intestinal epithelial cells from antigen exposure. In both the colon and small intestine, mucin 2 (MUC2) secreted by goblet cells is the most abundant mucin. MUC2 reduces antigen exposure to the immune system and intestinal epithelial cells, which is essential for disease prevention (13). Studies have shown that MUC2-deficient mice spontaneously develop colitis, and their mucus layer is more susceptible to bacterial penetration (14). Moreover, mucus defects are associated with the occurrence of neonatal Escherichia coli meningitis (15)and necrotizing enterocolitis (16), indicating that damage to the mucus layer can promote inflammatory responses, thereby inducing acute and chronic colitis as well as colorectal cancer (17). Beneath the mucus layer, intestinal epithelial cells (IECs) play a critical role in maintaining intestinal barrier integrity. Multipotent stem cells within the intestinal crypts differentiate into five cell types: absorptive enterocytes, goblet cells, enteroendocrine cells, Paneth cells, and microfold (M) cells (18). These cells form a continuous polarized monolayer that separates the lumen from the lamina propria. Molecular transport between IECs is regulated by intercellular junction complexes, primarily including tight junctions (TJs), adherens junctions (AJs), and desmosomes (19). TJs and AJs are connected to the surrounding actin-myosin network, thereby regulating intercellular adhesion through the cytoskeleton (11) (19),. Additionally, IECs express a variety of innate immune signaling molecules on their surface and within their cytoplasm (20), including intracellular peptidoglycan receptors NOD1 and NOD2, as well as surface and intracellular Toll-like receptors (TLRs), which recognize bacterial, fungal, and viral structures (21, 22) and activate immune responses via the NF-κB pathway (23). The lamina propria serves as the final line of defense in the intestinal mucosa, where immune cells interact with epithelial cells to collectively maintain intestinal barrier function. For instance, goblet cells deliver antigens to CD11c^+^/CD103^+^ dendritic cell (DC) subsets in the lamina propria (24), followed by cross-presentation of antigens by DCs, inducing the differentiation of Foxp3^+^ regulatory T cells (Tregs) (25, 26).

#### 2.1.2 Immunomodulation of the intestinal mucosa

Interferon-gamma (IFN-γ) and tumor necrosis factor-alpha (TNF-α), derived from T cells, are key mediators of intestinal inflammatory diseases, including inflammatory bowel disease (IBD). TJs and increase intestinal permeability by regulating the expression of claudin and occludin (27–30). Zolotarevsky et al. demonstrated that IFN-γ and TNF-α promote the redistribution of TJ proteins (ZO-1, JAM-A, occludin, claudin-1, and claudin-4) in intestinal epithelial cells (Caco-2 and T84), leading to impaired barrier function (31). This mechanism is likely mediated by myosin light chain kinase (MLCK), which facilitates TJ disruption through myosin light chain (MLC) phosphorylation. Inhibition of MLC phosphorylation can restore barrier function.

Th2 cell cytokines also play a role in regulating intestinal barrier function. Studies have shown that stimulation of colonic epithelial cell lines T84 and HT-29/B6 with IL-4 or IL-13 increases intestinal permeability (32–34). The underlying mechanism involves epithelial cell apoptosis and upregulation of claudin-2 expression. The PI3K pathway plays a critical role in this process (33, 35), as blocking IL-4/IL-13-mediated PI3K activation can prevent barrier dysfunction (33). IL-10, as an anti-inflammatory cytokine, also modulates intestinal barrier function (36, 37). Treatment with IL-10 can prevent IFN-γ-induced increases in epithelial permeability (38). Immune cells likewise contribute to the regulation of the intestinal mucosal barrier. For instance, CD3 stimulation leads to CD4^+^ T cell activation, resulting in increased permeability and enhanced secretion of IFN-γ and TNF-α (39, 40). Mice lacking intraepithelial lymphocytes (iIELγδ^+^) exhibit abnormal localization of claudin-3, occludin, and ZO-1, impaired TJ formation, and consequently, barrier dysfunction (41). Additionally, mast cells are widely distributed throughout the gastrointestinal tract (42). Upon activation, they release a variety of potent inflammatory mediators, including histamine, serotonin (5-HT), neutral proteases, prostaglandins, leukotrienes, platelet-activating factor, and cytokines such as TNF-α, IL-3, and IL-4 (43–45). Research indicates that mast cells participate in intestinal barrier regulation through models of food allergy or parasitic infection (46).

#### 2.1.3 Intestinal mucosal injury and related diseases

Intestinal mucosal injury can lead to diseases such as IBD, food allergies, celiac disease, and diabetes, with immune regulation being a key factor influencing its function (47). IL-10-deficient mice exhibit increased permeability and spontaneously develop chronic colitis (36, 48), indicating its protective role in barrier function. IL-10 may regulate intestinal permeability through the claudin/claudin receptor pathway and TNF-α-related mechanisms, and inhibition of this pathway has been shown to improve permeability and reduce the risk of colitis in IL-10-deficient mice (37). The zonulin/zonulin receptor pathway is believed to regulate TJ formation via PKC-dependent actin cytoskeleton remodeling (49).

Eosinophils and their granular proteins, such as major basic protein (MBP), eosinophil peroxidase, and eosinophil cationic protein (ECP), are increased in IBD and functional bowel disorders (50–53). *In vitro* co-culture experiments have shown that eosinophils or their major basic protein can reduce transepithelial electrical resistance (TER) in T84 cells, increase permeability, and downregulate occludin expression (54).

In summary, the function of the intestinal mucosal barrier is regulated by various immune cells and cytokines. Its damage can lead to a variety of intestinal diseases. A deeper understanding of its immune regulatory mechanisms will contribute to the development of related therapeutic strategies.

### 2.2 Respiratory mucosal barrier

#### 2.2.1 Composition and function of respiratory barrier

**Figure 2:**
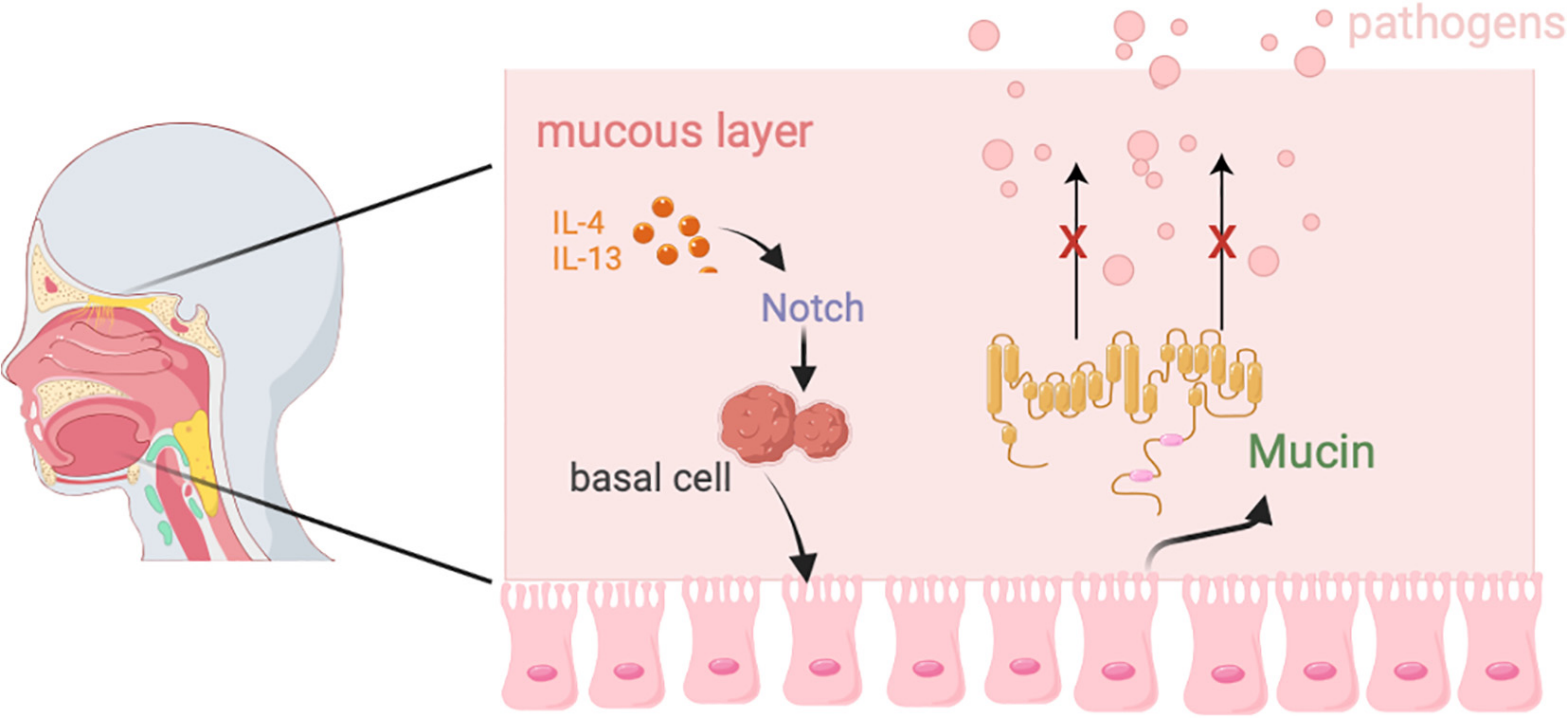
The composition and function of the respiratory barrier.

The respiratory tract is divided into the upper respiratory tract (URT) and lower respiratory tract (LRT). The URT includes the nasal cavity, pharynx, and larynx, which together form the pathway for air flow and contain associated lymphoid tissues such as the nasal-associated lymphoid tissue and cervical lymph nodes. The LRT includes the trachea, as well as the bronchi and bronchioles within the lungs (55). As a crucial barrier against external pathogens and particulate matter, the respiratory tract relies on the synergistic action of the mucous layer and epithelial cells to maintain functional homeostasis (**Figure 2**).

The respiratory mucosal barrier consists of a mucus layer and epithelial cells. The mucus layer is composed of an upper gel-like mucus layer and a lower ciliary surrounding layer (55), which forms a bottlebrush structure. Its main component is mucin, which is crucial for protecting the respiratory tract from pathogen infections (56). The respiratory epithelium is primarily composed of ciliated epithelial cells, goblet cells, basal cells, and club cells (57), with airway epithelial cells being central to the pathogenesis of major lung diseases, including COPD, asthma, and bronchial cancer (58). In these diseases, localized inflammation and immune signaling further impact the function of airway epithelium (59, 60).

#### 2.2.2 Regulation and function of epithelial cell differentiation

Ciliated epithelial cells are the predominant cell type in the airways, and their differentiation is strictly regulated by the conserved Notch signaling pathway (57). Inhibition of Notch signaling promotes the differentiation of basal cells into ciliated epithelial cells, while high levels of Notch signaling drive their differentiation into goblet cells (61, 62). Goblet cell hyperplasia and excessive mucus secretion are common pathological features of asthma, COPD, and cystic fibrosis. Research by Agrawal et al. suggests that blocking excessive mucus secretion in an asthma mouse model can alleviate airway obstruction (63). Although airway epithelial cell proliferation was not observed in the asthma mouse model, the data support the hypothesis of transdifferentiation from ciliated cells to goblet cells (64).

Goblet cells primarily secrete mucins, such as Muc5AC and Muc5B, to capture foreign molecules (65, 66).Under normal conditions, the production and clearance of these mucins maintain a dynamic balance. However, excessive differentiation of goblet cells driven by IL-4 and IL-13 disrupts this balance, leading to the development of asthma, allergic rhinitis (AR), and chronic rhinosinusitis (CRS) (67).

Basal cells are stem cell-like progenitors in the upper and lower airways. Under the regulation of the Notch signaling pathway, they can differentiate into ciliated epithelial cells, goblet cells, and other epithelial cells (68). They are tightly attached to the basement membrane and provide structural support through hemidesmosomes (69). Under homeostatic conditions, basal cells remain quiescent, but after barrier injury, they are rapidly activated and migrate to the damaged area to form a temporary barrier (70). Basal cell proliferation and differentiation are observed in chronic airway diseases. For example, studies on ex vivo basal cell cultures from nasal polyps in CRSwNP patients and bronchial biopsies from asthma patients have shown reduced proliferative capacity of basal cells (65). A recent study by Ordovas-Montanes et al. revealed that in CRS, the IL-4/IL-13 signaling signature persists in basal cells and their progeny, keeping airway epithelial cells in an undifferentiated state and reducing cellular and functional diversity. Blocking the IL-4 receptor α subunit can restore basal cell function (71).Additionally, basal cells can mediate innate immune responses. For instance, in cigarette-induced epithelial injury, they secrete antimicrobial protein RNase7 while upregulating innate immune mediators such as β-defensin-2, lipid-binding protein 2, IL-6, IL-8, and CCL20 (72, 73).

#### 2.2.3 Impaired barrier function and disease

Although the mucus layer plays an important role in the respiratory barrier, the true physical barrier is maintained by the adhesive complexes between epithelial cells, including TJs, adherens junctions, and desmosomes (65). TJs are composed of transmembrane proteins such as occludin, claudins, and the immunoglobulin-like (IgG) family members of JAMs, which are connected to cytoplasmic scaffold proteins like ZO-1, ZO-2, and ZO-3 (74). Adherens junctions are located beneath TJs and are formed by the E-cadherin-catenin complex (75). Desmosomes ensure the attachment of basal and other epithelial cells to the basement membrane (76). The epithelial cell barrier is closely associated with the development and progression of various respiratory diseases. Disruption of the epithelial barrier in allergic asthma is linked to TJ defects and reductions in adherens junctions and desmosomes (77). Compared to healthy controls, AR patients show reduced expression of occludin and ZO-1, which correlates with disease severity (78). Increased epithelial permeability and irregular, decreased expression of TJ molecules such as occludin and ZO-1 were found in the *in vivo* specimens of CRSwNP patients (79). Various cytokines have been shown to interfere with TJs, such as the typical Th2 cytokines IL-4 and IL-13, which are released upon allergen exposure and lead to epithelial barrier dysfunction (80). Steelant et al.’s study indicated that IL-4 and IL-13 disrupt the barrier integrity of nasal epithelial cells in both AR patients and healthy controls, resulting in a vicious cycle of increased epithelial permeability (81). In contrast, strengthening the epithelial cell barrier effectively reduces inflammation in various *in vitro* and *in vivo* models of Th2-mediated respiratory inflammation (82). Additionally, mediators released by mast cells can also increase epithelial permeability, making it easier for allergens to penetrate the host (83).

### 2.3 Urinary mucosal barrier

The urinary system consists of the bladder, ureters, and kidneys, primarily responsible for filtering and excreting waste while maintaining systemic homeostasis (84). The inner surface of the urinary tract is lined with a mucosal barrier composed of the urothelium, basement membrane, and lamina propria (LP) (85). The urothelium is a tightly packed transitional epithelium, with its surface covered by a glycocalyx composed of mucopolysaccharides, providing both physical and chemical protection to epithelial cells (86). Compared to the intestinal mucosa, the glycocalyx layer of the urothelium is thinner (87–90). Its main components include membrane-bound glycoproteins, glycolipids, and soluble factors such as galectins and proteoglycans (91). Glycosaminoglycans (GAGs) are linked to core proteins to form proteoglycans, with chondroitin sulfate and hyaluronic acid being the major components of the GAG layer, playing a crucial role in barrier formation and antimicrobial defense (91).

#### 2.3.1 Structure and function of the urothelium

The urothelium consists of three layers: the basal cell layer attached to the basement membrane, the intermediate layer, and the superficial or apical layer composed of “umbrella cells” (92). Umbrella cells are hexagonal and interconnect to form a dense barrier, strictly regulating the permeability of solutes and water through TJs, thereby effectively preventing harmful substances in urine from entering the tissue (92). Additionally, adherens junctions (AJs) and desmosomes between urothelial cells play a crucial role in maintaining epithelial integrity (93). Beneath the urothelium lies the LP, which is composed of an extracellular matrix and contains various cell types, including fibroblasts, interstitial cells, as well as afferent and efferent nerve endings (94). This structure not only provides mechanical support but also participates in signal transduction and immune regulation.

#### 2.3.2 Urinary tract microbiota and the infection mechanism

Urothelial injury and exposure to harmful substances may be associated with the pathology of spinal cord injury and are often accompanied by irritative lower urinary tract symptoms (95). Due to its anatomical proximity to the gastrointestinal tract, the bladder mucosa is frequently exposed to microorganisms. Additionally, in females, the urethral opening is close to the vaginal mucosa, resulting in a unique microbiota composition (96–98).

Urinary tract infection (UTI) is the most common infection worldwide, affecting either the upper urinary tract (pyelonephritis) or the lower urinary tract (cystitis). Uropathogenic Escherichia coli (UPEC) is the primary causative agent of UTIs (99). UPEC colonizes the lower gastrointestinal tract and can migrate across the perineum to the urethra, entering the urinary tract. It initiates UTI by covalently binding to the UPK1A protein expressed on the apical surface of umbrella cells via the adhesin FimH, located at the tip of type 1 fimbriae (100). Moreover, FimH can interact with urothelial cells to induce the exfoliation of umbrella cells, thereby promoting urothelial cell proliferation (101).

#### 2.3.3 Injury and repair of the urothelial barrier

Under homeostatic conditions, the mitotic activity of urothelial cells is largely quiescent, with a very slow cell cycle (102, 103). However, in response to acute injury caused by chemical exposure, surgical trauma, or urinary tract infection (UTI), urothelial cells rapidly proliferate to facilitate repair and regeneration (104). The urothelium expresses multiple Toll-like receptors (TLRs), which recognize pathogen-associated molecular patterns and damage-associated molecular patterns, thereby activating inflammatory responses and promoting the clearance of infected cells (105). The urothelial barrier plays a critical role in maintaining urinary tract homeostasis, preventing pathogen invasion, and facilitating tissue repair. Its barrier function primarily relies on tight junctions, the glycocalyx layer, and the structural support of the underlying lamina propria. Following UTI or injury, urothelial cells can swiftly initiate repair mechanisms, while TLR signaling pathways play a central role in inflammatory responses and pathogen clearance. Elucidating the regulatory mechanisms of the urothelial barrier will contribute to the development of effective therapeutic interventions for UTIs and related urinary tract disorders.

### 2.4 Oral mucosal barrier

The oral mucosa represents the initial segment of the gastrointestinal (GI) tract and shares anatomical and histological similarities with the GI system (106, 107). In addition to mucus secretion, the oral cavity produces saliva through the salivary glands (108, 109). Saliva is initially derived from serous exudates and is further enriched with a diverse array of molecules originating from mucosal cells, immune cells, and resident microbiota (109). The dynamic secretion and swallowing of saliva facilitate the mechanical clearance of pathogens (109). Moreover, saliva contains immunoglobulins (such as secretory IgA), antimicrobial peptides (such as defensins), and enzymes secreted by the salivary glands, collectively mediating innate and adaptive humoral immunity (109).

#### 2.4.1 Structure and immune function of the oral mucosa

Similar to other mucosal tissues, the oral mucosa consists of three structural layers: the epithelial layer, the lamina propria (LP), and specialized lymphoid tissue (106). However, unlike the single-layer columnar epithelium of the gastrointestinal tract, the oral mucosa is composed of stratified squamous epithelium, forming a thicker and denser mechanical barrier (106). The LP is a loose connective tissue rich in blood vessels and lymphatic vessels, serving as a primary site for the induction and effector functions of immune cells (110). Under homeostatic conditions, dendritic cells (DCs) are present throughout the lamina propria, freely migrating between self and foreign antigens (111) and playing a critical role in tolerogenic immune responses. Oral DCs typically express low levels of maturation markers (CD80, CD83, CD86) and exhibit high tolerance to stimuli from the oral microenvironment (112, 113). Upon encountering pathogens or injury, DCs become activated and migrate to lymphoid tissues, initiating T-cell immune responses (110). In mouse models, different subsets of CD11c^+^ DCs, along with Langerhans cells (LCs), are distributed within the epithelium of the oral, sublingual, and gingival mucosa. These antigen-presenting cells (APCs) capture antigens and deliver immune signals to T cells (114, 115).

#### 2.4.2 Oral mucosa-related diseases and immune regulation

Clinical studies have shown that the oral mucosa can be affected by various pathological factors, including viral infections, Candida infections, and OLP (110). Among these, immune deficiency and imbalance in the oral immune system are major predisposing factors for these diseases. Cases of mucosal immune dysfunction have been observed in individuals infected with human immunodeficiency virus (HIV). HIV infection leads to a reduction in CD4^+^ T cell levels, resulting in immune deficiency and making patients more susceptible to infections by commensal microorganisms in the oral and pharyngeal regions, such as Candida albicans (116).

Another case involves hyper-IgE syndrome, which is caused by mutations in STAT3 (117–119). Patients with hyper-IgE syndrome are highly prone to oral candidiasis due to the absence of TH17 cells, a finding consistent with animal studies: IL-17 receptor-deficient mice (IL-17RA^-/-^) and IL-23p19-deficient mice exhibit significantly increased susceptibility to C. albicans infection (120). Although TH17 cells are critical for combating oral fungal infections, their excessive activation may trigger chronic inflammation and even lead to autoimmune diseases (118, 121).

#### 2.4.3 Inflammation and autoimmune diseases of the oral mucosa

Under normal conditions, the immune response of the oral mucosa to food antigens and commensal bacteria does not induce inflammation but rather promotes immune tolerance. However, aberrant activation of the immune system may lead to inflammation and autoimmune diseases such as periodontitis, Sjögren’s syndrome, and OLP. Periodontitis is triggered by bacterial plaque accumulation, associated tissue damage, and bone loss due to the host immune response and inappropriate inflammation. T helper (TH) cells play a crucial role in the recruitment of neutrophils and osteoclasts, contributing to alveolar bone and gingival barrier destruction (122, 123).OLP is a chronic inflammatory disease characterized by massive lymphocyte infiltration in the LP and chronic destruction of the epithelial basal layer (124–126). Scully et al. demonstrated that TH1 and TH2 cells contribute to OLP-associated inflammation and mucosal lesion formation, with increased levels of pro-inflammatory cytokines, including IL-6, IL-17, and TNF-α, in the saliva and serum of OLP patients (125, 127–129). In contrast, serum levels of TGF-β are lower in OLP patients compared to healthy individuals (130, 131). A single nucleotide polymorphism (SNP) study on IL-10 polymorphisms revealed a higher frequency of four haplotypes (-1082 G/A, -819 C/T, and -592 C/A polymorphisms) in the peripheral blood of OLP patients, which is associated with reduced serum IL-10 levels (132).

Based on these findings, several reports suggest that T cells may be involved in the development of OLP (133). However, given that various immune cell types can produce these cytokines, the precise role of T cells in OLP pathogenesis remains to be determined.

## 3 Potential applications of natural products in mucosal barrier-related diseases

In recent years, natural products have received much attention in the study of mucosa-associated diseases due to their excellent safety, biocompatibility, and therapeutic potential (134, 135). Natural products can attach to mucosal surfaces, inhibit inflammation, modulate microflora, and enhance the expression of tight junction proteins (TJPs), thereby restoring intestinal barrier function (**Table 1**). Numerous studies have confirmed that polysaccharides can alleviate dextran sulfate sodium (DSS)-induced UC (136, 137).

**Table 1:** Mechanism of natural products and their extracts in the treatment of mucosal barrier related diseases.

Natural product	Compound Name	Chemokines or chemokine receptors	Machine	References
OBG	OBG-supplemented rats	High dose increases T lymphocyte ratio; low dose enhances claudin-3, claudin-4 expression	Regulates intestinal immunity, enhances barrier integrity	(139)
HSWP-1d	DSS-induced UC mice	Increases TJPs expression, balances pro-inflammatory/anti-inflammatory factors	Maintains intestinal barrier stability	(140)
LNT	CP-induced intestinal barrier damage in mice	Regulates IL-6, IL-2, IFN-γ, IgG levels; upregulates TNF-α, IL-1β, Occludin, ZO-1 expression	Reduces CP-induced intestinal damage	(141)
TFP	DSS-induced UC mice	Inhibits inflammation, restores intestinal and mucus barriers	Improves UC symptoms	(142)
CYP-1	DSS-induced UC mice	Inhibits colonic inflammation activation, restores TJPs expression, regulates gut microbiota	Maintains intestinal barrier function	(143)
APS	DSS-induced UC mice, RSL3-stimulated Caco-2 cells	Anti-ferroptosis effect	Improves experimental colitis	(144)
SPS	DSS-induced UC mice	Inhibits STAT3/NF-κB pathway, enhances TJPs and mucin expression	Maintains intestinal barrier integrity	(145)
XG	Allergic rhinitis mice	Reduces tissue damage, decreases pro-inflammatory factors, maintains ZO-1 expression	Protects nasal mucosal barrier	(146)
Matrine	Caco-2 cell model, DSS-induced UC mice	Mediates Rho-ROCK signaling pathway via miR-155, regulates ROCK1 expression	Maintains tight junction protein integrity	(150)
Hordenine	DSS-induced UC mice	Inhibits SPHK1/S1PR1 and STAT3 phosphorylation, improves goblet cell morphology	Reduces intestinal damage, enhances mucus barrier	(151)
IND	UC mouse model	Acts as an aryl hydrocarbon receptor (AhR) ligand, promotes CD4^+^ IL-10^+^ T cell proliferation	Enhances immunomodulatory function	(152)
INB	UC mouse model	Inhibits TLR4-mediated pro-inflammatory factor expression via NF-κB and MAPK pathways	Alleviates UC	(153, 154)
IND+INB	UC mouse model	Combined enhancement of intestinal barrier, synergistic action on AhR, NF-κB, and MAPK	Synergistically alleviates UC symptoms, improves therapeutic efficacy	(155)
Berberine	Acute and chronic UC mouse models	Regulates Th17/Treg immune balance, improves gut microbiota, enhances glial-epithelial-immune cell interaction	Alleviates colitis symptoms, restores intestinal homeostasis	(156, 157) (158)
Berbamine	DSS-induced UC mice	Mechanism similar to BBR, but effective at lower doses	Similar effects to BBR, but effective at lower doses	(160)
Saussurea costus	UC mouse model	Reduces TNF-α, IL-1β, IL-8, IL-18 levels, increases ZO-1 and Occludin expression	Improves UC pathological changes, enhances intestinal barrier function	(161)
Costunolide	DSS-induced UC mouse model	Binds to NLRP3’s Nacht domain, inhibits NLRP3 inflammasome assembly and ATPase activity	Significantly inhibits intestinal inflammation, exerts anti-inflammatory effects	(162)
Farnesol	UC mouse model	Reduces IL-6, IL-12, TNF-α, COX-2, INF-γ, increases IL-10 expression	Inhibits inflammatory response, reduces mucosal damage caused by leukocyte chemotaxis	(163–165)
OAG	Caco-2 cell model, DSS-induced UC mice	Enhances claudin-1, E-cadherin expression, increases TEER value	Enhances intestinal epithelial barrier function, maintains barrier integrity	(166)
Ori	DSS-induced UC mouse model	Inhibits intestinal mucosal cell apoptosis, reduces oxidative stress via Sirtuin-1/NF-κB/p53 pathway	Protects colonic mucosal barrier, reduces inflammation and oxidative damage	(167)
Ursolic acid	DSS-induced UC mouse model	Downregulates MAPKs, IL-6/STAT3, and PI3K classical inflammatory pathways	Delays UC pathological changes, such as weight loss and intestinal shortening	(168)
Paclitaxel	UC mouse model	Inhibits NF-κB signaling pathway and regulates gut microbiota	Effectively promotes recovery of intestinal barrier in colitis mice	(169)
Luteolin	IEC-18 cells and LPS stimulation	Inhibits IKK activity, blocks NF-κB signaling pathway	Downregulates pro-inflammatory gene expression, reduces intestinal epithelial cell inflammation	(175)
Naringenin	*In vitro* cell model	Protects IκB, prevents NF-κB translocation to the nucleus	Inhibits pro-inflammatory factor expression, reduces inflammation	(176)
Pomegranate juice	HT-29 cells + TNF-α treatment	Inhibits Cox-2 expression	Reduces TNF-α-induced inflammatory response	(178)
Red wine extract	HT-29 cells	Inhibits excessive IL-8 production	Reduces inflammatory factor release, alleviates inflammation	(179)
Marie Ménard freeze-dried apple	*In vitro* cell experiment	Reduces MPO activity, inhibits Cox-2 and iNOS gene expression	Reduces inflammatory factor release, alleviates inflammation	(180)
Flavonoid metabolites	DSS-induced colitis model	Downregulates TNF-α, IL-1α, IL-8 expression, reduces mucosal damage	Reduces inflammatory factor release, alleviates inflammation	(181)
Grape powder	Inflammatory colon cancer mouse model	Increases butyrate-producing bacteria abundance	Reduces inflammatory colon cancer incidence by 29%	(182)
Oat and bran polyphenols	DSS-induced enteritis mouse model	Regulates intestinal macrophages, inhibits T cell activation, enhances IL-10 expression, reduces TNF-α/IL-6 ratio	Restores gut microbiota balance	(183)
Quercetin	IBD mouse model	Regulates intestinal mucosal macrophage immune response via HO-1-dependent pathway	Balances gut microbiota, improves IBD	(184)
Arbutin	Ethanol and aspirin-induced gastric ulcer animal model	Enhances epithelial cell survival rate	Has gastric mucosal protective effects	(185, 186)
Arbutin	DSS-induced UC mouse model	Upregulates tight junction proteins (Occludin, Claudin, ZO-1) via MAPK/ELK1 pathway	Enhances intestinal barrier function	(187)
Kaempferol	DSS-induced UC mouse model	Regulates gut microbiota, downregulates TLR4-NF-κB signaling pathway	Improves intestinal permeability, reduces intestinal barrier damage	(188)
Formononetin	DSS-induced UC mouse model	Regulates gut microbiota, inhibits NF-κB signaling pathway	Protects intestinal mucosal barrier	(189)
Licochalcone A	DSS-induced UC mouse model	Inhibits apoptosis, maintains TJ expression, regulates gut microbiota	Maintains intestinal barrier integrity	(190)
Silibinin	Colitis-associated cancer mouse model	Inhibits STAT3 phosphorylation, inhibits IL-6/STAT3 signaling pathway	Reduces inflammatory factor production, alleviates intestinal mucosal barrier damage	(191)
Lactulose	UC mouse model	Downregulates inflammatory factors, regulates TLRs/NF-κB pathway, increases butyrate-related beneficial microbiota, improves intestinal mucosal barrier	Low-dose lactulose effectively improves UC symptoms, such as diarrhea, bloody stools, and weight los	(192)
HCA	UC mouse model/*in vitro*	Binds to STAT3, inhibits its activation and downstream signaling, reduces TJ damage, reduces apoptosis	Reduces UC-related intestinal barrier damage, reduces apoptosis	(193)
VA	IBD mouse model	Targets CA9, regulates INSIG2 and STIM1 interaction, inhibits ferroptosis-induced excessive apoptosis of intestinal epithelial cells	Alleviates IBD symptoms by regulating ferroptosis to inhibit excessive intestinal epithelial cell death	(194)
Daikenchuto	IBD mouse model	Increases abundance of beneficial microbiota such as Parabacteroides, Allobaculum, increases butyrate levels	Exerts anti-protease and anti-microbial activity	(195)
Inulin	IBD mouse model/*in vitro*	Induces β-defensin-1 and TJ gene expression in colon, improves intestinal permeability; fermentation produces SCFA butyrate and acetate	Alleviates IBD symptoms by inducing antimicrobial agent expression	(196–198)
FL3, FL37	Caco-2BBE and IEC-6 cells	Inhibits NF-κB and Cox2 expression, reduces inflammation, maintains mitochondrial survival, reduces cell permeability	Increases cell activity, inhibits inflammation, reduces intestinal barrier damage	(199)
Ginsenoside Rb1	UC mouse model	Downregulates pro-inflammatory factors TNF-α and IL-6, increases anti-inflammatory cytokine IL-10; upregulates TJ	Improves intestinal barrier by regulating immune factors and upregulating tight junction proteins	(200)
VK2	UC mouse model	Reduces pro-inflammatory cytokine levels, increases IL-10 expression, promotes mucin and tight protein expression	Improves UC symptoms	(201, 202)
FHL	UC mouse model	Induces macrophage M2 phenotype differentiation via Notch signaling pathway	Alleviates colonic inflammation	(203)
QBD	UC mouse model	Inhibits inflammatory cascade via NF-κB and Notch signaling pathways, improves intestinal permeability	Improves colitis symptoms in UC mice	(204)

### 3.1 Polysaccharides

Magdalena et al. found that dietary oat β-glucan (OBG) supplementation modulated gut immune responses and barrier integrity. Rats receiving high-dose (3%) OBG exhibited a higher percentage of LP T lymphocytes, whereas those receiving low-dose (1%) OBG showed significantly increased expression of intestinal barrier proteins claudin-3 and claudin-4 (138, 139).

Ni et al. extracted a mannose-glucan (HSWP-1d) from Hirsutella sinensis, which effectively improved DSS-induced colitis symptoms in mice and maintained intestinal barrier stability by enhancing TJP expression and regulating the balance of pro-inflammatory and anti-inflammatory factors (140). Likewise, Jin et al. evaluated the protective effects of lentinan (LNT) on cyclophosphamide (CP)-induced intestinal barrier injury by assessing serological markers, histopathological changes in ileal tissues, TJP expression, and cytokine levels. The results indicated that LNT significantly alleviated CP-induced abnormalities in body weight, immune organ index, and serum IL-6, IL-2, IFN-γ, and IgG levels (p<0.05), while increasing the mRNA levels of TNF-α, IL-1β, IFN-γ, occludin, and ZO-1 (p<0.05), thereby mitigating CP-induced intestinal barrier damage (141).

Furthermore, Tremella fuciformis polysaccharides (TFP) have been shown to exert therapeutic effects in DSS-induced UC models by suppressing inflammation and restoring intestinal and mucus barrier functions (142). A water-soluble polysaccharide (CYP-1) from Dioscorea opposita inhibited the activation of colonic inflammation, restored TJP expression, and regulated gut microbiota in UC mice (143). Astragalus polysaccharides (APS) have been demonstrated to ameliorate experimental colitis in DSS-challenged mice and RSL3-stimulated Caco-2 cells, significantly inhibiting ferroptosis (144). Additionally, safflower polysaccharides (SPS) alleviated intestinal inflammation in UC models by suppressing the STAT3/NF-κB signaling pathway, protecting goblet cells, and enhancing the expression of TJPs and mucins, thereby improving intestinal barrier integrity (145).

Moreover, polysaccharides offer advantages in restoring barrier integrity with fewer adverse effects, ultimately improving patients’ quality of life. Marika et al. evaluated the therapeutic efficacy of xyloglucan (XG) nasal spray compared to several standard treatments (corticosteroid sprays, oral mast cell stabilizers, and oral antihistamines) for AR. The results indicated that XG exhibited significant efficacy in reducing histological damage in AR mice, suppressing pro-inflammatory cytokines, and maintaining ZO-1 expression (146). Additionally, xyloglucan has been shown to possess barrier-forming protective properties in adult and pediatric gastroenteritis and dry eye symptoms, making it a safe, non-pharmacological alternative for various diseases (147). Its potential role in other mucosal barrier disorders, such as dermatological diseases, warrants further investigation.

### 3.2 Alkaloid

Alkaloids are important active compounds in natural herbal medicines, characterized by highly diversified heterocyclic structures (148). Among them, plant alkaloids have attracted attention in traditional Chinese medicine due to their anti-inflammatory properties, which can suppress the expression of pro-inflammatory cytokines, lipid mediators, histamine, and inflammation-related enzymes (149). Based on this, alkaloids are considered important candidate drugs for repairing the mucosal barrier. Yu et al. studied the protective effect of matrine on the intestinal barrier through miR-155 in the Caco-2 cell line, DSS-induced colitis in mice, and clinical samples from patients with obstructive sterility. The results indicated that matrine could promote the expression of ROCK1, a protein associated with the Rho-Rock pathway, in Caco-2 cells and maintain tight junctions (150). Xu et al. treated DSS-induced ulcerative colitis (UC) mice with hordenine, and the histological examination showed that intestinal damage in the treatment group was significantly improved. Additionally, compared to the control group, the hordenine-treated group exhibited a more regular arrangement of goblet cells, more complete cell shapes, and a greater surface coverage of glycoproteins and other mucous substances, showing a certain dose-dependent effect. At the molecular level, hordenine inhibited the increase in sphingosine kinase-1 (SPHK1) and sphingosine-1-phosphate receptor-1 (S1PR1) expression, as well as the phosphorylation of STAT3 in the colon tissue of DSS-induced mice (151). Indirubin (IND) and indirubin-3’-monoxime (INB) are isomers and active molecules of natural indigo in traditional Chinese medicine, with therapeutic activity against UC. IND is a ligand for the aryl hydrocarbon receptor (Ahr), which can promote the proliferation of CD4^+^ IL-10^+^ T cells (152). INB reduces the expression of inflammatory factors such as TNF-α and IFN-γ through the NF-κB and MAPK signaling pathways mediated by TLR4, thereby eliminating inflammation (153, 154). Xie et al. explored the therapeutic effect of combined IND and INB treatment for UC, and the results showed that this combination could synergistically enhance the function of the intestinal barrier (155). Previous experiments have shown that berberine (BBR) can alleviate acute and chronic experimental colitis by regulating the T17/Treg balance (156), gut microbiota balance and metabolism (157), and the interactions between gut glial cells, epithelial cells, and immune cells (158). Dong et al.’s proteomics study indicated that the Wnt/β-catenin pathway was significantly enhanced in the colon tissue of mice treated with BBR, and the therapeutic effect of BBR was lost after intervention with the Wnt pathway inhibitor FH535, suggesting that BBR protects the mucosal barrier through the Wnt/β-catenin pathway (159). Interestingly, berberine’s main active metabolite, berberrubine (BB), has also been shown to exert a similar effect in attenuating DSS-induced UC, with a similar mechanism but at much lower doses (160).

### 3.3 Terpenoid

Terpenoid compounds are widely present in various traditional Chinese medicines and have been shown to repair intestinal barrier function by downregulating inflammatory factors and increasing the expression of tight junction proteins. Pang et al. revealed that Saussurea costus could reduce the levels of TNF-α, IL-1β, IL-8, and IL-18, while enhancing the expression of ZO-1 and Occludin, thereby improving the pathological characteristics of ulcerative colitis (UC) (161). They identified its main components, including proline, phenylalanine, isoleucine, ganoderic acid M, and pyroglutamic acid. Xu et al. demonstrated that the main active ingredient of Saussurea lappa, the sesquiterpene lactone Costunolide (COS), exerted a potent anti-inflammatory effect in a UC mouse model by inhibiting the NLRP3 inflammasome. Although the specific mechanism is not fully elucidated, the study suggested that COS could bind to the Nacht domain of NLRP3, altering its ATPase activity and inflammasome assembly (162). Farnesol (FAR), one of the main volatile oil components of grapefruit flowers, is a natural sesquiterpene alcohol (163). FAR alleviates intestinal inflammation and reduces intestinal mucosal damage caused by leukocyte chemotaxis by lowering the levels of IL-6, IL-12, TNF-α, COX-2, and IFN-γ, while increasing the expression of IL-10 (164, 165). Wang et al. demonstrated in Caco-2 cell models and DSS-induced mouse models that oleanolic acid 28-O-β-D-glucopyranoside (OAG), a naturally occurring pentacyclic triterpene, enhances intestinal epithelial barrier function by increasing the expression of tight junction proteins (claudin-1 and E-cadherin) and raising TEER values (166). Wang et al.’s research also showed that oridonin (Ori) could alleviate DSS-induced UC inflammation in mice and reduce oxidative stress levels, while inhibiting intestinal mucosal cell apoptosis through the Sirtuin-1/NF-κB/p53 pathway, thereby protecting the integrity of the colonic mucosal barrier (167). Sheng et al. demonstrated that ursolic acid (UA) downregulated three classical inflammatory signaling pathways—MAPKs, IL-6/STAT3, and PI3K—effectively delaying weight loss and intestinal shortening in mice (168). Hou et al. showed that dietary paclitaxel effectively enhanced the recovery of the intestinal barrier in colitis mice by inhibiting the NF-κB signaling pathway and regulating the gut microbiota (169).

### 3.4 Flavonoid

Flavonoids have been shown to exert protective effects on the epithelial barrier (170). Studies suggest that the underlying mechanisms may be closely related to the regulation of tight junction proteins (TJs) and the balance of the gut microbiota. For example, cranberry extract, which is rich in flavonoids, significantly increases the proportion of Akkermansia spp. in the mouse gut (171). Akkermansia, a mucin-degrading bacterium in the intestinal mucus layer, has been confirmed to be crucial for maintaining epithelial integrity (172). Luteolin, a flavonoid abundant in plants such as carrots, peppers, and celery (173, 174), has been shown by Jin et al. to inhibit the activity of IκB kinase (IKK) in LPS-induced IEC-18 cells (175), thus blocking the NF-κB signaling pathway and reducing the expression of pro-inflammatory genes. Additionally, flavonoids such as naringenin can protect IκB from degradation, preventing the translocation of NF-κB to the nucleus and further inhibiting the expression of pro-inflammatory factors (176). Activated NF-κB is involved in the transcription and activation of genes related to immune and inflammatory responses, such as pro-inflammatory cytokines (TNF-α, IL-1β, and IL-6) (177) and inflammation-related enzymes (Cox-2 and iNOS). Flavonoids play a significant role in suppressing intestinal inflammation and the expression of pro-inflammatory enzymes. In one study, pretreatment of HT-29 cells with pomegranate juice rich in anthocyanins and catechins resulted in a reduction of TNF-α-induced Cox-2 expression (178). Nunes et al. observed that pretreatment with red wine extract containing catechins, oligomeric procyanidins, and anthocyanins effectively suppressed excessive IL-8 production in HT-29 cells (179). Furthermore, naringenin can downregulate adhesion molecules (ICAM-1), chemokines (MCP-1), iNOS, Cox-2, TNF-α, and IL-6 (176). Marie Ménard’s freeze-dried apples, rich in flavonols and flavan-3-ols, reduce myeloperoxidase (MPO) activity and inhibit the expression of Cox-2 and iNOS genes (180). MPO is considered a marker of disease activity in intestinal inflammation, further supporting the regulatory role of flavonoids in intestinal inflammation.

It is important to note that the anti-inflammatory properties of flavonoids in the intestine may be mediated by their metabolites. After entering the intestine, flavonoids are metabolized by intestinal cells and the microbiota, resulting in a series of bioactive metabolites. A study by Larrosa et al. demonstrated that certain flavonoid-derived metabolites significantly inhibit DSS-induced colonic mucosal damage and downregulate the expression of TNF-α, IL-1β, and IL-8 (181). However, research on flavonoid metabolites is still limited, and it is expected that this will become a key direction for elucidating their mechanisms of action and developing novel anti-inflammatory drugs in the future.

### 3.5 Polyphenols

Polyphenolic compounds are primarily found in plant-based foods and regulate the gut microbiome through dynamic interactions with intestinal microbes, alleviating intestinal inflammation and enhancing gut barrier function. Zhao et al. discovered that, compared to a normal diet, the inclusion of polyphenol-rich grape powder in the diet reduced the incidence of inflammatory colon cancer in mice by 29%, with the mechanism related to the increased abundance of butyrate-producing bacteria in the gut (182). Duan et al. demonstrated that the polyphenols in oats and wheat bran could regulate intestinal macrophages, inhibit T-cell activation, enhance IL-10 expression, and significantly reduce the TNF-α/IL-6 ratio, thereby restoring gut microbiota balance (183). Quercetin (QCN) can regulate the immune response of intestinal mucosal macrophages through a heme oxygenase-1 (HO-1)-dependent pathway, improving gut microbiota imbalance and alleviating IBD (184). Studies have shown that arbutin protects against ethanol- and aspirin-induced gastric ulcers in animal models (185) and increases epithelial cell viability (186). Zhang et al. demonstrated that arbutin mediates the expression levels of tight junction proteins (Occludin, Claudin, and ZO-1) through the MAPK/ELK1 signaling pathway (187). Qu et al. used a UC mouse model to show that kaempferol improves intestinal permeability and significantly prevents DSS-induced intestinal barrier disruption by regulating gut microbiota and downregulating the TLR4-NF-κB signaling pathway (188). Similarly, Peng et al. demonstrated that astragaloside can improve the intestinal mucosal barrier function in DSS mice by reducing gut microbiota dysbiosis and inhibiting the NF-κB pathway (189). Zhang et al. revealed that Glycyrrhiza chalcone A (LA) maintains intestinal barrier integrity by inhibiting cell apoptosis and maintaining TJ expression. Additionally, 16S rRNA analysis indicated that LA also regulates gut barrier-associated microbiota (190). Zheng et al. found that silymarin significantly inhibited the phosphorylation of STAT3 in colitis-associated cancer (CAC) mice, thereby suppressing the IL-6/STAT3i signaling pathway to reduce the production of inflammatory cytokines and alleviate damage to the intestinal mucosal barrier (191).

### 3.6 Other extracts from natural products

The study by Cui et al. demonstrated that low-dose lactulose effectively alleviates symptoms of UC, including diarrhea, hematochezia, and weight loss. Its mechanism involves downregulation of inflammatory factors, modulation of TLRs/NF-κB signaling pathways, and reduction of cecal pH, promoting the proliferation of beneficial microbiota such as SCFAs, thereby improving the intestinal mucosal barrier (192). Chen et al. discovered that the natural product 2-hydroxycinnamaldehyde (HCA), isolated from cinnamon bark, directly interacts with STAT3 to inhibit its activation and downstream signaling. This compound effectively mitigated UC-induced disruption of intestinal barrier tight junctions both *in vitro* and *in vivo*, reducing apoptosis and improving intestinal inflammation (193). Ni et al. showed that vanillic acid (VA) targets carbonic anhydrase IX (CA9), facilitating the interaction between INSIG2 and STIM1, which promotes SCAP-SREBP1 translocation and activates SREBP1. This enhances SCD1 transcription, inhibits ferroptosis, and prevents excessive intestinal epithelial cell death (194). Some natural products exert anti-inflammatory effects by modulating the gut microbiota and regulating microbial metabolites. Daikenchuto (DKT) prevents IBD by exhibiting anti-protease and anti-microbial activity via secretory leukocyte protease inhibitor (SLPI), increasing the abundance of butyrate-producing bacteria such as Parabacteroides, Allobaculum, and Akkermansia, thereby enhancing butyrate levels (195). Inulin and sodium butyrate also improve intestinal permeability by inducing β-defensin-1 and tight junction proteins. SCFAs generated from inulin fermentation further stimulate antimicrobial peptide expression by Paneth cells (196–198). *In vitro* results indicated that flavonoids FL3 and FL37 increased basal activity of Caco-2BBE and IEC-6 cells, reduced apoptosis, and decreased epithelial monolayer permeability. Their mechanism involved the suppression of TNF-α and IFN-γ induced NF-κB and COX-2 expression to alleviate inflammation. Additionally, they preserved mitochondrial survival by maintaining complex I activity and inhibiting TNF-αinduced mitochondrial superoxide generation (199). Zhou et al. used RNA-seq and network pharmacology to study ginsenoside Rb1 in UC, finding that it downregulated TNF-α and IL-6 while increasing IL-10 and tight junction proteins (ZO-1, Occludin, E-cadherin). These effects may be linked to VDR, PPARγ, and NF-κB signaling pathways (200). Vitamin K2 (VK2), a naphthoquinone derivative, has been shown to worsen UC symptoms in mice fed a vitamin K-deficient diet (201). Hu et al. demonstrated that VK2 reduces pro-inflammatory cytokine levels, increases IL-10 levels, and promotes the expression of mucins and tight junction proteins to restore mucosal barrier function (202). Additionally, traditional Chinese medicine formulas have shown potential efficacy. For example, Huaihua Decoction regulates the Notch signaling pathway in mice, inducing macrophage M2 phenotype differentiation, thus enhancing intestinal barrier function in DSS-induced colitis (203). Qingbai Decoction (QBD) similarly modulates NF-κB and Notch signaling to inhibit inflammatory cascades and enhances the mucus and epithelial cell barriers, improving intestinal permeability in colitis mice (204).

The gut microbiota plays a key role in mucosal repair. UC patients often show reduced levels of SCFA-producing bacteria. Zhao et al. found that supplementing mice with SCFA-producing bacterial supernatants restored SCFA levels and inactivated the JAK/STAT3/FOXO3 axis, leading to M2 macrophage polarization and improved colonic health (205). Zhuang et al. demonstrated that extracellular vesicles from O. splanchnicus effectively alleviated weight loss, colon shortening, disease activity index, and histological damage in a DSS-induced IBD mouse model. These effects were associated with the downregulation of IL-1β, TNF-α, and IL-6 expression, the upregulation of IL-10, and the blockade of NLRP3 inflammasome activation, thus ameliorating intestinal barrier dysfunction and colonic apoptosis (206). Yue et al. reported that cytoplasmic membrane vesicles (CMVs) secreted by L. reuteri, which interact with host cells, were taken up by intestinal epithelial cells. This uptake enhanced the expression of ZO-1, E-cadherin, and occludin, reduced intestinal permeability, and improved tight junction function, thereby alleviating DSS-induced colitis in mice (207). Pan et al. evaluated the preventive effect and mechanisms of Lactobacillus fermentum 016 (LF) in a DSS-induced UC mouse model, showing that LF improved intestinal mucosal barrier function through the Nrf2-Keap1 signaling pathway and modulation of systemic inflammatory factors such as IL-1β, IL-6, TNF-α, IFN-γ, IL-4, and IL-10 (207). Cui et al. also showed that HnAg (membrane shell antigen) intervention in UC mice increased goblet cell numbers and elevated mucin and tight junction protein expression. These effects were likely mediated through activation of the AhR/IL-22 pathway (208).

## 4 Discussion

The mucosal barrier is widely present in various organ systems, including the gastrointestinal tract, respiratory tract, urinary tract, and oral cavity. As an important defense line against harmful external substances, it performs multiple functions such as physical barrier, immune defense, and microbial balance. The integrity of the barrier is maintained by structures such as tight junctions, adherens junctions, and desmosomes between epithelial cells, and it works in conjunction with abundant immune cell populations to form the mucosal immune system, which regulates inflammatory responses and immune tolerance (209).

However, various pathological factors, such as infections, inflammation, autoimmune abnormalities, environmental toxins, drugs (such as NSAIDs and antibiotics), and poor dietary habits, can disrupt the mucosal barrier, leading to increased barrier permeability (leaky mucosa), which in turn triggers IBD, ulcerative colitis (UC), Crohn’s disease (CD), respiratory diseases (such as asthma and chronic obstructive pulmonary disease, COPD), gastric ulcers, oral ulcers, and other conditions (210). These diseases are typically accompanied by chronic inflammation and may further progress to cancer, such as colorectal cancer (CRC) and esophageal cancer. Therefore, repairing the damaged mucosal barrier has become a crucial strategy in the treatment of these diseases (211).

Currently, conventional treatment options for mucosal barrier injury-related diseases mainly rely on chemical drugs, including aminosalicylates, corticosteroids, immunosuppressants (such as azathioprine and cyclosporine), and biologics (such as TNF-α inhibitors). Although these drugs can effectively control inflammation and alleviate symptoms in the short term, long-term use may lead to a range of side effects, such as immunosuppression, osteoporosis, hyperglycemia, and liver and kidney damage. Furthermore, these treatments typically target a single pathway and are insufficient in comprehensively addressing the complex pathophysiology of the diseases. Additionally, some patients may develop drug resistance or poor therapeutic response. Therefore, identifying safer treatment strategies with broader mechanisms of action has become a research focus.

In recent years, extensive studies have shown that natural products offer advantages in improving mucosal barrier damage, with multiple targets, high safety, and fewer side effects, showing promising therapeutic effects in refractory diseases (210). Moreover, the wide variety of traditional Chinese medicine provides abundant sources for treating mucosal barrier diseases. The main mechanisms of natural products in mucosal barrier injury diseases include the regulation of immune cell functions, reduction of inflammatory factor levels, and promotion of tight junction protein expression to aid in mucosal barrier repair and restore its function (212).

With the deepening research on microbiota, the modulatory effects of natural products on the microbiome are gradually being revealed. For instance, some natural products can increase the abundance of short-chain fatty acid-producing bacteria, thereby enhancing the levels of butyrate in the gut (213). Butyrate can be absorbed by intestinal epithelial cells and assist in mucosal barrier repair. Microbial-targeted therapies have become a novel strategy for mucosal barrier repair. Additionally, combination therapy with natural products exhibiting complementary mechanisms of action can improve therapeutic efficacy, providing ideas for exploring new treatment options.

Although natural products demonstrate significant potential in the treatment of mucosal barrier diseases, several challenges remain. For example, current research mainly focuses on gastrointestinal mucosal repair, while studies on mucosal tissues in other organs, such as the respiratory, oral, and urinary systems, remain limited. Furthermore, some traditional Chinese medicines or formulations, although showing good efficacy, still lack a clear understanding of their specific mechanisms of action and active components, requiring further investigation. The interactions between natural products and the microbiome also warrant deeper research to fully elucidate their mechanisms and enhance clinical applications.
